# Resilience in the Vaccine Supply Chain: Learning from the COVID-19 Pandemic

**DOI:** 10.3390/vaccines13020142

**Published:** 2025-01-29

**Authors:** Megan Hay, Anika Teichert, Sarah Kilz, Agnes Vosen

**Affiliations:** Fraunhofer Center for International Management and Knowledge Economy IMW, 04109 Leipzig, Germany; megan.hay@imw.fraunhofer.de (M.H.); digital.health@imw.fraunhofer.de (S.K.); agnes.vosen@imw.fraunhofer.de (A.V.)

**Keywords:** vaccine supply chain, vaccine production, mRNA, resilience, COVID-19, environmental analysis

## Abstract

**Background:** The COVID-19 pandemic revealed vaccine supply chain (VSC) weaknesses and enabled post-pandemic analysis highlighting the growing importance of supply chain resilience. This study analyzes weaknesses and potentials for VSC resilience from an industry perspective. Insights from this study are aimed at supporting helping managers and policy-makers build a more resilient vaccine supply. **Methods:** A qualitative semi-structured interview study was conducted with 12 industry experts along the VSC. The interviews were assessed concerning the learnings from the pandemic in a two-step content analysis. Codes were assigned to key VSC concepts and variables and then linked to political, economic, social, technological, legal, and environmental (PESTLE) dimensions. The complex multi-stakeholder supply chain was visualized in a system overview, highlighting main actors, roles, constraints, and resilience. **Results:** The analysis resulted in 60 codes, categorized into the six PESTLE dimensions and three additional (sub)groups (mRNA, Supply chain resilience, and Solutions). The largest dimension was Economic, with 39 codes, including the Supply chain resilience subgroup. Twelve stakeholder groups were identified, with purchasers, manufacturers, suppliers, developers, and regulatory agencies being the most significant in emergency vaccine manufacturing situations. **Conclusions:** The system overview demonstrated the VSC as a complex network of actors with unaligned goals rather than a linear supply chain. This study shows that the VSC is characterized by uncertainty due to external factors, like the unpredictability of new emergencies, and internal factors like vaccine demand. The lack of transparency between industry stakeholders exacerbates VSC disruption. We conclude that infrastructures and management practices that enable increased transparency and collaboration between stakeholders hold the greatest potential for strengthening the VSC’s resilience to future pandemics.

## 1. Introduction

The COVID-19 pandemic was a health crisis with enormous socio-economic effects worldwide that required rapid action and resilient leadership [1]. During COVID-19, time was also of the essence in the development, production, and distribution of life-saving vaccines, which are an immensely important preventive measure that provides protection from infection to individuals, and cost-efficiency to health systems, contributing directly to the UN’s Sustainable Development Goal 3: Good Health and Well-being [2,3,4]. Historically, the development of a new vaccine has taken over ten years on average due to extensive testing, highly regulated clinical trials, and high failure rates [5]. In order to accelerate this process in the critical pandemic situation, these normally successive steps in clinical trials were parallelized, and mass production was ramped up even before the official licensing of vaccines [6]. Next to traditional vaccine technology, the COVID-19 messenger RNA (mRNA) vaccines were the first of their kind to gain approval of the European Medical Agency (EMA) [6], harboring a large potential for rapid adaptation to new viruses and strains due to their expected ease of modification [7]. As a result, the first (emergency-)licensed vaccines were administered to high-risk groups after just ten months of development [8].

The VSC is an extensive network beginning with the procurement of raw materials and continuing to distribution and waste management [9], whereby disruption in any step can have cascading effects on the global vaccine supply [10]. Global travel, urbanization, climate change, infringement on animal habitats, and shortage of health workers are all factors contributing to the increasing risk of pandemics [11]. For this reason, we must learn from the supply chain issues arising in the COVID-19 pandemic to be prepared for future health emergencies, producing and distributing a new vaccine quickly and equitably. For the smoothest possible vaccine rollout, all parts of the VSC must be operable [10]. Uncertainty presents a challenge because it is deeply characteristic for the pandemic VSC landscape and extends from overarching pandemic management strategy by governments [12,13] to technical decisions in development [14,15] to supply-chain operations management [16,17,18]. The market uncertainties manifest, for example, in actual consumer demand being impossible to measure accurately and subject to a lack of information and misinformation [13,18,19]. Additional uncertainties for companies arise out of competition within the industry [13,18], as well as pending regulatory outcomes of new vaccine candidates [15,20]. Especially for novel technologies such as mRNA vaccines, the technological process and regulatory parameters were not entirely clear [7,14,21]. For companies, all these uncertainties translate to financial risk; in disregard of this, VSCs are generally geared towards efficiency [16,22,23]. To identify the potential for improving VSCs in future pandemics, we apply a theoretical framework consisting of three parts: leagility, Theory of Constraints, and resilience.

Traditional supply chain models aim for efficiency to save costs [24], while excessing capacities constitute a cost factor. The epitomical example of efficient supply chains is the automotive industry, in which parts are ideally delivered “just-in-time” to production, thus minimizing the necessary storage efforts [25,26], which may be also desirable in other industries, and enabling greater production flexibility through planned redundancies. This combination of lean and agility—leagility—provides a framework for balancing efficiency and agility in a business supply chain based on the characteristics of the marketplace [26]. Applying this to vaccines, the supply chains for routine vaccinations can be quite lean since demand is stable and predictable and the product has a long life cycle [26]. Conversely, new vaccines that are meant to impact the dynamics of an ongoing pandemic, like the COVID-19 vaccines, require a more agile supply chain, though this agility is limited by long lead times in vaccine production [24].

Continuous process improvement can be sought by identifying existing constraints in the supply chain network and restructuring the organization accordingly, thereby applying the Theory of Constraints (TOC) to supply chain management. Alleviating system-limiting factors can lead to a higher level of performance or output through efficient utilization and should not be confused with a bottleneck—a resource that is not available to meet demand during the period [25]. These constraints, which include those arising from managerial politics [25], could change the development of relationships with other actors in the supply networks, such as the level of trust that enables strategic data sharing for joint planning, forecasting and replenishment, thus improving the transparency of supply [26]. During a global health crisis and national lockdowns, TOC can be applied to (re-)assess where the supply network can be lean and where redundancies should be incorporated for agility. The high-volume production and distribution of a new vaccine can interfere with the capacities of associated networks, e.g., those for routine vaccines, due to the complexity of the VSC networks [27]. TOC is well-established in supply chain management and more recently has found applications in the realms of healthcare and resilient pharmaceutical supply chains [25,28,29,30]. TOC thus relies on system thinking, which acknowledges the dynamic interrelations between systems [25,31], expanding upon the binary leagility concept and making it operable on the dynamic systems that are at the base of VSCs.

The term resilience has its origins in the field of psychology, describing the response of a system to a disturbance [32]. There is no universally agreed-upon definition of resilience within management studies; however, in this study, the working definition of Radic et al., 2022 is used:

“*We define business model resilience as the ability of an organization to sustain its value proposition despite unexpected current and future disruptions […]. This ability can manifest at the individual level, team level, organizational level, or environmental level*” [32].

**Figure A1:**
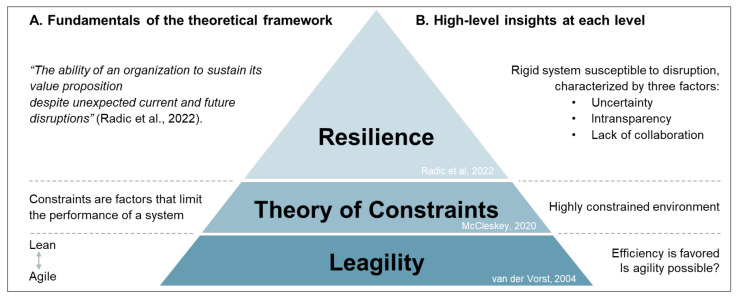
This figure illustrates the theoretical framework created for this study. (**A**) The framework is based on three published and widely used frameworks, that build on each other in complexity from binary (leagility), delimited (Theory of Constraints), to a complex systems perspective. (**B**) The high-level insights of this study at each conceptual level are summarized in brief.

Resilience aims to design the system to best cope with and recover from unforeseen disruptions [32]. Resilient business models are complementary to risk management, which tends to focus on mitigating foreseeable problems [32]. Trump et al. distinguish between businesses installing resilience measures and external resources such as governments or insurances doing so [33]. In Radic et al.’s 2022 study of pandemic impacts on the manufacturing industry, supply chains emerged as the factor most severely affected by the COVID-19 pandemic [32]. In a pandemic, strategically installed redundancies could ensure delivery and thus contribute to business model resilience; at the same time, these redundancies constituted a cost factor in the absence of disruptions [32]. Taken together, VSCs are a critical resilience lever in pandemic management, where the many stakeholders in the system are subject to distinct sets of constraints. The theoretical framework for this analysis of the VSC is based on three concepts, building on each other in complexity—from a binary (leagility), to a delimited (Theory of Constraints), to a complex systems perspective (see Figure A1). Leagility and Theory of Constraints are long-established and widely used [25,26,31]. Novel in this study is the expansion with the systems perspective, which enables the rationalization of indirect and knock-on effects in terms of system resilience [32].

In the face of increasing risks of pandemics and the challenges in the development, production and distribution of life-saving vaccines experienced during the COVID-19 pandemic, this study analyzes weaknesses and potentials for VSC resilience from an industry perspective [10,11]. The implementation of novel mRNA vaccines merits an additional focus due to the perceived potential for acceleration of the pandemic response. The aim is to identify vulnerable business areas in the VSC that require better preparation or reorganization. Given the high importance of stakeholders and their collaboration during the pandemic, this study presents the central stakeholders in vaccine production during the COVID-19 pandemic and embeds these in the VSC system context. The results contribute to the knowledge on pandemic preparedness from a manufacturing perspective, which emerged from qualitative interviews with experts approached along the VSC.

## 2. Materials and Methods

**Figure A2:**
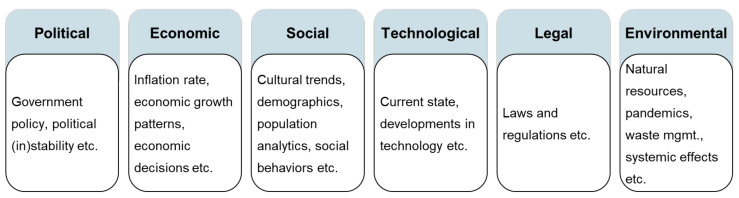
PESTLE dimensions, adapted from [35,36]. The political dimension includes, for example, government and political (in)stability. Economic factors encompass inflation rates, economic growth patterns and economic decisions. They have a direct impact on the economic capacity of a company/a market [35]. Social factors consider the social context such as cultural trends, demographics, population analytics, and social behaviors. The technological analysis looks at the developments in technology, automation, and others. Legal factors consider for example laws and policies. And the environmental factors are impacted on by natural resources, pandemics, waste management, or systemic effects [35,36].

This study analyzes the insights from expert interviews and discusses the current barriers and potential solution approaches for improved VSC resilience. Beforehand, in September 2022, a systematic literature analysis on resilience in VSCs was conducted using the Web of Science database for English-language results to identify weaknesses in the supply chain for vaccine production under disruption and potential solutions. Since resilience is rather new in its application to economic topics, the query “supply chain*” AND “vaccin*” was used, focusing on more recent approaches since 2010. The remaining publications were examined for relevance, and the full-text versions were assessed. Results were classified into PESTLE dimensions to understand the external environment and its impact on the VSC for new vaccines using a qualitative content analysis [34]. PESTLE analysis is a common tool used to assess each dimension of the macro environment in which a company operates [35]. Fornasiero et al. applied this to supply chain studies, where these factors can be used by managers to characterize the status of the supply chain and their impact on the future development of their business [36]. For a description of the PESTLE dimensions based on [35,36], refer to the appendix (Figure A2).

To complement theoretical knowledge with practical experience, a qualitative interview study with experts approached along the VSC was conducted, building a case study on resilience in the VSC based on real-world experience during the COVID-19 pandemic. Due to the dynamic interrelations between systems, a system-level analysis was used, directing the focus to vaccine manufacturers, yet we also included their upstream partners, downstream partners, as well as overarching institutions (Table 1). A variety of stakeholders were included in the interview study to capture a holistic portrayal of VSCs. Participants were affiliated with stakeholder groups either in manufacturing itself or with up- or downstream actors. Professionals were approached via publicly available e-mail contact information that was researched on the internet, as well as via the contacts of persons associated with a project called “Intelligente Medizin” at Fraunhofer Gesellschaft, in which potentials for accelerating vaccine development along the value chain were identified and summarized in a concept paper [37].

To adapt the questions to the different roles of the participants and to gain possible additional information, a semi-structured interview format was chosen. The overarching structure of the interview usually followed the following themes: (1) VSC system-level questions, making a distinction between the “normal” situation and the “pandemic” situation, and (2) supply-chain resilience considerations. Participants were supplied with a high-level overview of the interview guide and informed about the data protection policy, and their consent was obtained. The main questions were posed to all participants, while the probes differed based on their role and field. The questions in the interview guide refer to the theoretical framework to ensure internal validity. The nine independent interviews with 12 experts along the VSC were conducted between November 2022 and January 2023 either in English (*n* = 4) or in German (*n* = 5). The interviews, which lasted between 30 and 60 min, took place via Microsoft Teams with two researchers and, with the consent of the 12 interviewees, were recorded and automatically transcribed using the Microsoft Teams transcription feature. Transcripts were corrected and anonymized manually by referring to the recording before analysis by one researcher. The Section 3 refers to interview participants where possible without compromising anonymity.

**Figure A3:**
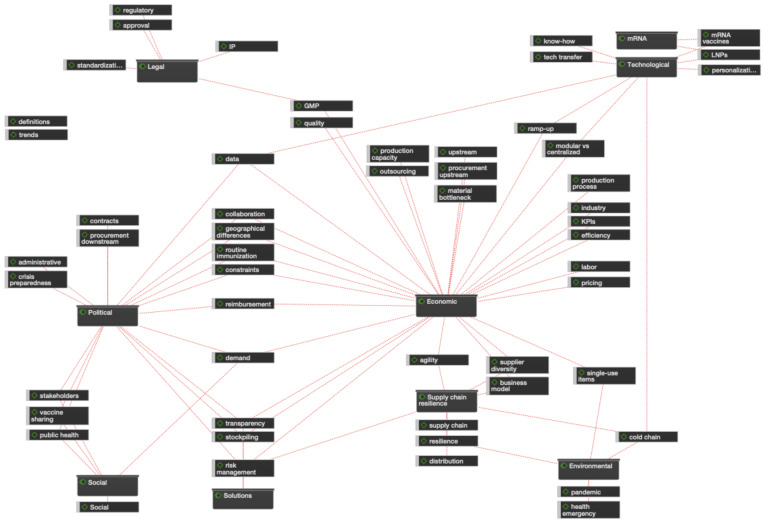
The code network shows the codes that emerged from analysis of the interview data. Note that there were codes with the same label as PESTLE dimensions for quotations relevant to the dimension that did not have an obvious unique code; these codes belong to the respective PESTLE dimension and are not depicted for simplicity.

To analyze the protocols, a two-step iterative thematic analysis of the interview data using the ATLAS.ti software Version 22.2.3 was conducted, similar to the methodology in Fornasiero et al.’s extensive study on the future of supply chains [36], which includes one researcher and a focus group consisting of two researchers from the field of digital health and focuses on socio-economic research. In the first step, codes of important concepts and variables of the VSC when raised by participants were applied and represented in a code network. An open coding approach was used for the purpose of capturing practical industry knowledge. This inductive approach allows the interview data to guide the analysis and is useful for highlighting participants’ perspectives [38]. Regarding information saturation, we followed the advice of Guest [39] and could assume data saturation after analyzing all 12 interviews. The results were discussed and validated by a focus group. Adapting the methodology from Fornasiero et al., PESTLE analysis was leveraged to link the codes under the respective aggregate dimensions, which provided an expansive framework for structuring the results and evaluating the VSC landscape (see Figure A3). This ensured that all the external factors in the macro environment could be captured. The transcripts were not returned to the participants for correction or comment, and no feedback on the results was obtained.

In analyzing the interviews, we aimed for a holistic perspective with system-level questions, with a subsequent deep dive into VSC resilience.

## 3. Results

### 3.1. Literature Review

The previous literature review identified approaches to analyze VSCs ranging from literature reviews and analyses to interview and modeling studies. The search returned 631 results, of which 25 were considered after checking the relevance and full-text versions. The VSC landscape was examined and issues that are central to resilience in VSC were identified, particularly in relation to vaccine production. The focus was on the political, economic, and technological dimensions of the VSC. Resilience terminology is not commonplace in the literature but has recently gained prominence, and there are efforts to create a common language for the different academic fields involved [40]. Although not all publications use resilience terminology, most of the articles reviewed explicitly address VSC at the system level or at least recognize significant impacts on the associated networks.

### 3.2. Code Network from Interview Data

In this section, the interview data are presented, starting with the code network showing the codes that emerged from analysis of interview data (see Figure A3). The number of codes emerging from the conversation, as well as their quantity, is used as a semi-quantitative measure to evaluate the relative importance of different topics. Arranging of codes to the PESTLE dimensions clarified that the largest dimension was Economic (39 codes including subgroup Supply chain resilience), followed by Political (19 codes), Technological (12 codes), Legal and Environmental (6 codes each), and finally Social (5 codes). We added the code group Solutions to easily identify the mention of solutions, without the intention of modulating the PESTLE framework. Out of 60 codes, the 10 most frequently occurring codes were “stakeholders”, “supply chain”, “constraints”, “pandemic”, “business model”, “solutions”, “production capacity”, “regulatory”, “geographical differences”, and “approval”, constituting over 40% of the total code occurrences (see Table A1). These codes aided in the identification of central aspects relevant to the efficiency and resilience of the VSC.

### 3.3. Stakeholders in the VSC

**Figure 1:**
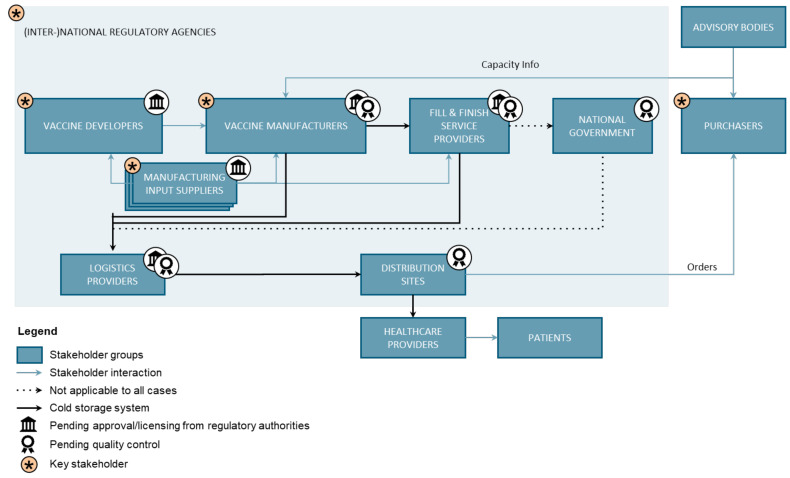
System-level representation of the stakeholders and interactions in the vaccine supply network. The illustration shows the most important relationships in the system. Deviations by country and vaccine type are possible [9].

The various stakeholder groups and their collaboration during the COVID-19 pandemic emerged as crucial to create and sustain supply. Therefore, the results presented in the following focus on the key players in vaccine production, particularly vaccine purchasing, supply and manufacturing, because we aim to provide evidence for the decision-making of managers and policy-makers. These are embedded in the broader context of the stakeholder network to identify weaknesses and opportunities for more resilient VSCs. Disease outbreaks are not confined by borders, and the VSC is highly interconnected globally; thus, the system overview created in this study intentionally transcends borders. Twelve stakeholder groups and their interactions in the VSC were identified from literature research and interviews, and the results are presented by each of the five stakeholder groups that emerged as particularly important for vaccine production in emergency situations: purchasers, manufacturers, suppliers, developers, and (inter)national regulatory agencies (Figure 1). In the following, the main actors, their role and drivers, the constraints within which they operate, and considerations and potential solutions for VSC resilience are illustrated. In the explanation of the results, references are made in brackets to the participant (P#) who made the statement in the interview (see Table 1). The findings detailed in the stakeholder overview are summarized to provide a convenient overview (Table A2), demonstrating a rather intricate network of actors with often unaligned goals, which may impede efficient collaboration. To fare well in extraordinary situations such as a pandemic, preparedness involves better defining roles and drafting solutions for foreseeable conflicts of interest.

#### 3.3.1. Purchasers

The entities that procure vaccines are, depending on country and situation, national governments; supranational authorities (e.g., European Commission); non-governmental organizations that procure on behalf of many governments, like in rather low-income countries (e.g., GAVI, UNICEF, UN agencies); or the private sector (pharmaceutical wholesalers, and in rare cases individuals).

The respective public health system determines which vaccines are made available to the population, with large differences between countries (P4—for details on participants, see Table 1), while non-governmental vaccine markets are more common in wealthier countries (P9, P11). In both cases, the purchasers are not the end users themselves, and instead purchasers must increase demand for the vaccines by raising awareness and reducing costs (P5, P7). In the pandemic scenario, as exemplified by the COVID-19 pandemic, governments take a central role beyond procurement in the VSC across geographies by investing in vaccine development and facilitating partnerships with and between manufacturing stakeholders (P3, P4). Governments and NGOs both may engage in facilitation and matchmaking between public and private players, as well as tracking distribution and immunization progress since they have a vested interest in the efficient and safe handling of the purchased vaccine (P5). The interviewees of the government entity for pandemic management mentioned that the drivers of the vaccine market are generally public health and disease control; thus, the common goal is the provision of safe and effective vaccines to the target population (see Table 2).

The effectiveness of the pandemic VSC was constrained by a lack of transparency along the VSC, starting from the availability of input materials (P4, P9) and no comprehensive overview of the geographical sources of materials and production capacities (P9) to understanding and locating demand (P5), impeding the ability to plan. The actual capacity for manufacturing was an acute bottleneck (P3, P4), and subsequently, capacity for the transport of vaccines both depended initially on the level of existing infrastructure and workers’ expertise (P5). While the demand in wealthy countries was very high, significant quantities of COVID-19 vaccines only reached low-income countries after the first wave had passed (P5). Together with ubiquitous misinformation, the demand had dropped, and despite the infrastructure finally being available, it proved difficult to identify where the demand was and distribute vaccines accordingly (P5). Three interviewees mentioned that during the COVID-19 pandemic, both the United States and the European Union released implicit or explicit export controls to favor their own production and delivery of COVID-19 vaccines (P4, P6, P7). This impeded other nations’ ability to procure the desired amount of vaccines (P6, P7). Countries that do not produce vaccines, which tend to be low-income countries, were entirely dependent on the import of vaccines, so the contracts between vaccine manufacturers and wealthier countries caused great delays in access to COVID-19 vaccines in poorer regions of the world (P5). When left unchecked, the market often does not incentivize the development of vaccines against the pathogens that most threaten public health (P10, P12). The financial risk of producing a vaccine pending market authorization is so high that governments or other purchasers must correct this market failure by mitigating the risk and providing advance funding to ramp up production (P3, P4, P12).

If the industry cannot resolve shortages itself, the purchaser can assist by connecting stakeholders, and governments can take policy measures to redirect supply favorably (P3, P7). Especially in low-income countries, regional solutions are the only way to increase local engagement and acceptance of purchased vaccines by the target population, for which knowledge of demand is crucial to prevent vaccine wastage (P5). Many countries would benefit greatly from being less dependent on vaccine imports by building local manufacturing capacity, but the necessary technology transfer will take years (P5). In preparation for future health emergencies, a better overview of the VSC and risk levels of different sources is necessary (P4, P9, P10). Governments especially could instill resilience into the system by stockpiling vaccines in preparation for outbreaks and reserving “warm” production capacities at manufacturers, which would immediately be available surge capacity in emergencies (P3, P9). At the time of the interviews, this was already being negotiated, e.g., in a tender for warm production capacities of the European Commission in 2022 (P3, P11), [41]. This also means that purchasers with reserved capacities automatically have more VSC capacity information at their disposal (P9).

#### 3.3.2. Vaccine Manufacturers

Vaccine manufacturers encompass all parties involved in their production, including CMOs, to which several production steps are outsourced (P1, P7, P8), consisting of several actors for different production steps. Typically, further distinction is made between manufacturers producing vaccines on a smaller scale for clinical trials and large pharmaceutical companies such as Pfizer and Sanofi (see Table 3).

The manufacturers’ role is the production of drug products (the active pharmaceutical ingredient) and drug substances (formulation with potential adjuvants, excipients, buffers) while complying with good manufacturing practices such as working towards the highest quality and safety standards (P1, P4, P12). Their main drivers are the demand for the vaccine and companies’ business interests (P1, P6, P7). Overall, the vaccine industry is experiencing growing demand, driven by the higher need of the growing population, a higher social acceptance of vaccines, and a higher awareness of the risk posed by pathogens (P7).

Although long-term planning is common in vaccine production, supply shortages regularly occur, even in routine production (P6, P7, P8, P9), including the effect of the frequent shipping between the individual production steps (P1, P4). In the case of the COVID-19 pandemic, material shortages were the most acute problem for the sudden ramp-up of the enormous production capacities in the billions of doses (P3, P4, P6). This was true especially for disposables and some raw materials that require qualification (P1, P4, P7, P8). If a supplier is unable to deliver, the manufacturer must first validate and qualify a new product and a new location to be able to change the supplier, which severely limits the flexibility of manufacturers due to the time and cost expenditure involved (P4, P7). Production also depends on the on-time delivery of the various input materials from a large number of suppliers (P6, P7). Some respondents see their decision to qualify additional suppliers or not as a trade-off between efficiency and flexibility (P6, P7), while others reject flexibility as a feasible measure in the pharmaceutical supply chain (P1, P4), with little to no flexibility built into the vaccine manufacturing supply chain (P1, P6, P7). Prior to the COVID-19 vaccine ramp-up, much of the CMOs’ capacity was reserved, so capacity was not readily available (P6). Vice versa, the capacity acquired for COVID-19 vaccine production may also reroute resources for the production of other medical products (P6). As each batch had to be tested after each step, both in-house and by laboratory partners who were over capacity during the pandemic, quality control took up most of the time in the production process (P1, P8). Vaccine production took only 70 to 100 days during the COVID-19 pandemic, whereby contamination could bring production at a particular site to a halt for months (P3). The increasing distribution of production steps to separate sites had advantages for the quality of the individual production steps but entailed risks during transportation and a dependency on the functionality of the geographically distributed sites (P1, P4, P7). The transfer of knowledge and technology to new facilities and CMOs in the production network required time and skilled labor (P3, P4), resulting in challenges in establishing the complex production processes faced especially by new players during the pandemic. The growth of the industry was expected to lead to difficulties in securing skilled labor and had already done so in some locations (P3, P7), with both technically skilled and academic workers becoming scarce (P4). Travel restrictions led to further delays in technology transfer during the pandemic (P4). Experiences differed as to whether intellectual property concerns were further slowing down this process (P4, P8, P12). Overall, pharmaceutical companies typically shared as little data as possible to protect their business interests (P4, P6, P8). During the COVID-19 pandemic, however, the necessity for collaboration became apparent, with one manufacturer attributing its business continuity during the pandemic to its trusting relationships with strategic suppliers and associated transparency regarding production necessities (P8).

Due to the required qualification of the products and locations, the production was bound to delivery from this specific source, resulting in purchasing from a single source (P1, P4, P6, P8). However, stockpiling would tie up capital and would only be feasible on a small scale and at short notice (P8), so agility was often not an option. The possibility or even a regulatory obligation to qualify products according to technical characteristics rather than the supplier’s brand would improve the flexibility of manufacturers with multiple suppliers (P4, P6, P7) and thus allow for more standardization of input materials (see Table 3). Prior to the pandemic, pharmaceutical production relied heavily on outsourcing (P1), resulting in fragmented networks of subcontractors who, prompted by the disruption, considered moving activities back in-house to have more control over promised deliveries and greater security (P7). Manufacturers were also looking to shorten and regionalize their supply chains, because this reduces transportation and the associated costs and risks (P1, P3, P6). Strategic goods, which are not easily replaceable, constitute an expectation in that manufacturers aim to qualify dual or triple sources of supply for these crucial items, preferably from distinct geopolitical areas in case of disruptions (P6, P8). Participants envisioned a supply chain optimized for security as a global supply network with locally established players (P6, P8). Leveraging technological solutions like digital twins of production and supply could create a real-time, transparent overview of production needs (P1, P10). The production of mRNA vaccines always requires the same quite-small volumes of input materials (which are few compared to traditional vaccines (P1, P8, P12)), whereby only minimal factors require tweaking to adapt the vaccine to another target (P2, P12), the manufacturing process is always the same and personnel that are trained once can be employed anywhere with this setup (P12). The sequence of RNA can be changed quite simply to represent a different antigen (P12). There are high expectations that this could enable additional time-savings in the regulatory process once the platform technology for mRNA is well-developed (P12). Taken together, this lends itself to a modular design of production, where drug substances can be produced quickly with high yield. However, there are immense differences between the mRNA manufacturing and traditional vaccine production, so existing know-how in the industry was scarce (P3), as was equipment for the LNP formulation and specific fill-and-finish capacities (P4). In addition, there is a high risk for contamination in mRNA vaccine production (P6) and the need for ultra-cold storage (−80 °C) to ensure stability could pose a challenge in regions where the electricity network is unreliable (P5).

#### 3.3.3. Manufacturing Input Suppliers

This stakeholder group encompasses raw materials suppliers, vaccine capital equipment suppliers, and fill-and-finish capital equipment suppliers, while a single vaccine manufacturer may rely on several hundred separate suppliers (P4, P6, P8). The context of vaccine equipment suppliers, whether large companies like Merck Sharp and Dohme and Cytiva or smaller companies, is particularly relevant and will be a focus of this section (P4, P6, P8), [9]. The market is trending towards higher consolidation (P6).

Their respective roles are the supply of raw materials: cell cultures and media, buffers, and chemicals; vaccine capital equipment—mainly single-use items like bags, filtration units and bioreactors; fill-and-finish capital equipment—materials for vial-filling like glass vials and stoppers, syringes and needles [15,42]; and also cold storage systems [7,10]. Around 70% of vaccine capital equipment tended to be custom-made for each manufacturer (P6). The main driver was a business interest based on demand from vaccine manufacturers and developers (see Table 4). Suppliers aim to keep lead times as short and as stable as possible (P6). Adherence to quality was imperative in this industry, and cost-efficiency was important to maintain a competitive market position (P6).

Lead times were constrained by capacity utilization, and during scarcities caused by the COVID-19 pandemic, component availability became a limiting factor. Despite these constraints, joint planning with vaccine manufacturers was undertaken with a focus 24 months into the future (P6). Vaccine producers often communicated demand only after vaccine purchaser negotiations had been concluded (P6). Due to the increased demand during the COVID-19 pandemic, demand could not be met in acute phases (P6, P8), exacerbated by some manufacturers abusing their prioritization due to COVID-19 vaccines to build up large safety stocks (P6, P7, P8). Political initiatives to prioritize local demands both in the United States and Europe further impeded the ability of suppliers to deliver to customers abroad (P4, P6, P7, P8). Considering that a lot of vaccine capital equipment was produced in the United States, European suppliers experienced a lot of pressure to fill this gap overnight (P6, P8). Once the capacities at the direct (tier-1) suppliers were shored up after the first demand shock, the bottleneck moved deeper into the supply chain to the tier-2 and -3 suppliers as vaccine production efforts relied on the same input materials (P6). After the spike of demand, it dropped quickly, resulting in financial difficulties for the small tier-2 and -3 supplier companies (P6, P8).

Suppliers could have prepared better for the sudden changes if scenarios for the expected demand had already been shared by manufacturers during the negotiations of contracts (P6). The standardization of input materials such as disposables reduces manufacturers’ dependence (P4, P7), has potential for the modularization of production, which could reduce changeover times and heighten the level of automation (P6, P7), and would also bring more flexibility and cost savings to the supplier (P6, P7). Less transport is more sustainable and shortens lead times, while large actors could balance demand with other plants as necessary for resilient supply. Deeper into the supply chain, complete regionalization is currently not feasible, nor is it desirable, since it preserves international cooperation (P6). Regulators and pharmaceutical companies showed more flexibility during the pandemic and a higher awareness for supply safety in practice, presenting the opportunity for lasting changes (P6, P8). Overall, information gathered by a third party with no business interests should shed light on the true demand and provide guidance on which production truly is essential and therefore must be prioritized (P6).

#### 3.3.4. Vaccine Developers

Apart from large pharmaceutical companies, academic and biotech companies are also often involved in the development of new vaccines [22].

Development precedes production for the masses, and the central driver is the development of safe and effective vaccines to protect the population from harmful pathogens (P12), while the safety and side-effects profiles of vaccines are held to the highest possible standards before they gain regulatory approval (P1).

The research and development efforts for a successful vaccine are enormous, since clinical trials for vaccines require very large participant groups in the tens of thousands, rather than hundreds—as is customary for therapeutic medicines (P1, P12). For completing the trials, however, smaller developers like academic and small biotech companies will generally need a large pharmaceutical company as a partner or buyer (P12). Due to the market potential of the vaccine in combination with the large clinical trials, vaccine development is a very expensive investment over several years that holds high economic risks (P1, P12). Accordingly, the main constraint is the uncertainty of regulatory approval of a high-risk investment (see Table 5).

Alleviating the risk of development can thus prevent market failures and correct the activities towards producing vaccines, also for pathogens with a lesser profit potential, for which developers already receive high amounts of funding from national governments and NGOs (P12). The perceived threat of viral diseases heightened during the COVID-19 pandemic (P7, P12), as evidenced by the increase in the importance of vaccinations and stockpiles for disease prevention on the political agenda (P7).

#### 3.3.5. International and National Regulatory Agencies

The steps of vaccine production are carried out under strict regulations, with many areas having their own regulatory bodies, such as the European Medicines Agency (EMA) or the Food and Drug Administration (FDA) of the United States, as well as the equivalents in Japan, Singapore, Switzerland, Israel, and China (P1, P5). The WHO conducts a “prequalification” for vaccines, whereby the quality, safety and efficacy standards are confirmed if successful and the vaccine is declared acceptable for procurement by UN organizations [43].

Regulatory authorities are responsible for ensuring the safety of medical products by reviewing the results of clinical trials, issuing market authorizations and approving production processes, production sites and suppliers for commercial production (P1) [44]. National regulatory authorities are responsible for inspecting vaccines imported from other countries, so a regulatory authority can send inspectors to a site in another country if that site changes supplier (P1). Industry and regulatory authorities want to speed up the time to market of medicines, and major improvements have been made in the last ten years (P1, P6, P8, P11).

Reviewing market applications for vaccines requires time, and developers and manufacturers have to bear the costs of applying for market authorization or qualifying sites and suppliers (P6, P7), with rigid procedures requiring a lot of paperwork (P1, P4, P8). The regulators themselves are constrained by upholding high safety standards and the time necessary to review all materials (P4, P8).

The documents and evidence required to approve a medical device are not uniform across the various regulatory bodies, which complicates procedures for developers and manufacturers (P1). During the COVID-19 pandemic, regulatory decisions have been accelerated by, for example, emergency use authorizations (FDA) or rolling review procedures (EMA), meaning the continuous review of evidence compared to the regular procedure (P1). Vaccine developers and manufacturers did not identify any lasting changes in regulatory processes by the time of the interviews; however, the flexibility shown during the pandemic presents an opportunity for continuous dialog with government agencies in future vaccine development (P6, P8). Regulators are recognized by industry experts for their progressive stance and progress in improving the system without compromising the safety of vaccines (P1, P6, P8). Regulatory agencies could create more flexibility for production through the standardization of input material qualification based on technical specifications and not on supplier brand, which could benefit both supplier and manufacturer (see Table 6). Further potential lies in digital tools to enhance regulatory and manufacturing processes and to speed up and facilitate the dialog between industry and regulators (P1, P10).

## 4. Discussion

**Figure 2:**
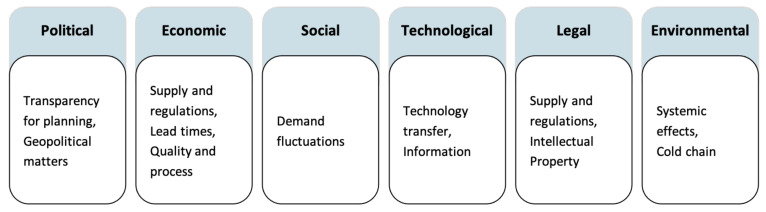
High-level overview of constraints identified from interviews by PESTLE dimension.

To relate the results back to the VSC landscape, the system constraints from individual stakeholder groups are mapped back to the PESTLE dimensions (Figure 2). This high-level overview illustrated the overarching constraints that govern a rapid ramp-up of vaccine production capacities, including those for new technologies such as the mRNA vaccines in the COVID-19 pandemic. In this section, the interview results regarding the concepts of agility, Theory of Constraints and resilience form the basis for the discussion (see the high-level summary in Figure A1B).

### 4.1. Leagility

The balance between efficiency and agility (leagility) is a foundational supply chain concept [26]. While the literature laments a lack of flexibility in capacity-building for vaccines [42,45,46], from the industry perspective, opinions diverge on whether the concept of agility applies to the VSC. Some participants engage with agility in their work and aim to increase agility, especially in the procurement of input supplies (P6, P7), while from other perspectives, the high standards of the regulatory framework simply do not allow for agility, as they are necessary and should not be lowered (P1, P4). Throughout the VSC, stakeholders’ ability to be agile is especially restricted by the extensive regulation of vaccines, as well as by the financial costs of installing redundancies in the VSC (P4, P6, P8) [24]. With the exception of regulators, all actors strive to optimize efficiency under normal circumstances; nevertheless, industry experts agree that single-sourcing is not likely to function well under disruption (P6, P7, P8). Regulators have the agency to change the “rules of the game” by promoting the standardization of input materials; this would improve supplier automation, manufacturer flexibility, and downstream handling efficiency without threatening vaccine safety (P4, P6, P7), [22,46].

### 4.2. Theory of Constraints

In the interview results, the most acute constraint mentioned was the physical bottlenecks, from raw materials, single-use items, vials and packaging to the capacities of logistics providers or actual vaccination centers (P3, P4, P6, P8), and [12,13]. While it might be possible to reduce the severity of bottlenecks through keeping strategic items in stock, the elimination of initial physical bottlenecks does not appear feasible. For one, stockpiling is only economically viable in the short term (P8), and secondly, some items may only become strategic once the situation unfolds (P8). For instance, no one could have predicted that mRNA vaccines would be approved for COVID-19 and be produced in such gigantic volumes. Since this vaccine type was not on the market, no reasonable enterprise would have stockpiled the mRNA production materials. However, much of the essence for receiving materials during the acute shortages in the early stages of the COVID-19 pandemic was the trust and relationships between manufacturers and suppliers (P8). During the pandemic, business relationships with a trust basis enabled the business partners to share more detailed information than usual, which allowed for the distribution of scarce supplies to the most critical producers and saved the system from an inefficient gridlock (P8). A major threat here was those companies that used their involvement with COVID-19 manufacturing to gain priority and build large stockpiles of up to two years’ worth of material, which drew material away from where it was most needed and exaggerated the demand, affecting suppliers negatively in the aftermath (P6). Moreover, various secondary and tertiary effects of diverting such large capacities to the COVID-19 VSC arose, such as taking up capacity for the production of other medical products and financial issues of small suppliers (P6). There was no official guidance in place describing which other vaccines and other life-saving drugs capacities were essential and could not be halted in favor of the pandemic response (P6). An increased distribution of one vaccine can lower the stock levels available for another [27], which, together with restricted movement and decreased access to other vaccines, led to the decline in routine immunizations registered during the COVID-19 pandemic (P5).

### 4.3. Resilience

Vaccines are complex products relying on numerous input materials [10] and are therefore at a high risk of disruption. The industry’s focus on efficiency combined with rigid regulatory processes results in a constrained system (as detailed in the section on leagility). This is not conducive to a resilient response to disruption, because it discourages two approaches to install more resilience in the system: stockpiling and diversifying suppliers. Stockpiles could address foreseeable disruptions; however, they constitute a cost factor in a system optimized for efficiency [24]. Diversifying suppliers is discouraged due to the costs and efforts associated with the qualification of materials as well as their suppliers, which is required by regulatory agencies (P4, P7).

At the same time, external factors may enhance the perceived benefit of installing resilience. We see that external factors like the growing likelihood of infectious disease outbreaks [11] and other catastrophes (P4) are exacerbating the vulnerabilities of the landscape within which the VSC lies. With an increased likelihood of large-scale disruptions that deeply impede not only business continuity but also public health, installing some strategic redundancies while accepting the increased costs becomes more convincing (P6, P7, P8). Depending on the business position, a company may benefit from government support, which externalizes the costs of business continuity onto society, or they may not receive support, which may render business non-viable (P6). Currently, the industry is returning to the pre-pandemic status quo of pharmaceutical manufacturing (P7, P8), with a growing awareness that the status quo is no longer sufficient to maintain its value proposition in a volatile environment (P6), [46], so there is nonetheless a general willingness among industry and policy stakeholders to create a more resilient system (P6, P7).

The importance of organizational knowledge became clear; thus, we recommend that businesses build an exhaustive knowledge base and carefully curate a workforce that contributes to and implements this knowledge, while CMOs can greatly complement the internal knowledge with highly specialized capabilities (P1). The right balance is important because inefficiencies can arise from the lack of oversight in the fragmentation of processes through excessive outsourcing [22]. In addition, the development of digital tools for regulatory matters and manufacturing processes harbors high resilience potential (P1, P10). In the VSC, these tools were not yet as sophisticated as in other industries; often the automotive sector is mentioned as a front-runner on their implementation (P6). Tools such as real-time digital twins could provide a more accurate foundation for strategic business decisions that need to be made in dynamically unfolding situations like a pandemic [45,47].

The business, cultural or regulatory surroundings are very strict in the VSC, where every step performed in the process must adhere to highest standards. In the pandemic scenario, vaccine business is also closely tied to collaboration with governments and thus transcends into the geopolitical constraints that companies must consider in their strategic choices (P7, P11). In the VSCs for different COVID-19 vaccines, the levels of outsourcing varied greatly. Nevertheless, a common theme was the emergence of geographically parallelized supply chains, as illustrated by the emergence of VSCs during the COVID-19 pandemic [22]. A clear trend towards shortening and regionalizing supply chains was corroborated in the interviews (P1, P3, P6). For large actors, a global network of regional supply chains was feasible and greatly ameliorates geographical and geopolitical risks (P6, P8). Smaller stakeholders were likely to confine their operations to their own geopolitical sphere (P8). Due to the experience during the COVID-19 pandemic, manufacturers contemplate at minimum identifying potential additional sources and service providers much earlier, and at best already qualify more than one supplier for strategic products to begin with (P6, P7, P8). Additional measures for supplier diversity, supply-chain shortening (such as the in-house assembly of certain manufacturing input materials), and the stockpiling of strategic items do constitute cost factors, yet they are necessary steps to ensure business continuity in the face of future disruptions (P8). Finally, efforts set on building trusted relationships construct the foundation for transparency with business partners (P8), [25]. In a crisis context, the ability to collaborate transparently within their immediate network proved to be a decisive factor in ensuring business continuity in the VSC (P6, P7, P8). Societal awareness of the benefits of immunization had increased overall (P7), though not without a degree of polarization and misinformation (P5). Misinformation about incidence rates, prevalence, and treatment options, as well as a public distrust of science and the rapid approval of vaccines, led to vaccine hesitancy, among other things. This also has an impact on sustainability because, for example, expired vaccines generate waste. Interested readers can read more about this in the following sources: [48,49,50,51]. Misinformation leads to people acting uncooperatively (e.g., vaccine hesitancy) and thus to physical (e.g., drinking disinfectant) and mental health impacts [49]. Indisputably, there is potential for more transparency in the agreements between governments and manufacturers, which would be conducive both to planning for suppliers and increasing public trust (P6). Furthermore, more understandable in-depth information on the development process, as well as the benefits and necessity of vaccinations, can contribute to educating society, e.g., using social media (and engaging influencers), clarifying misinformation and conducting community workshops [49,51]. From the industry perspective, the measures outlined in this section should be taken to build resilience into the VSC by design (concept from [33]). To optimize health outcomes for the population, there was a clear need for intervention by an overarching authority to ensure resilience (P3, P4, P7, P9, P11, P12). In most cases, government authorities were likely to assume this role (P4, P11). Government funding enabled several companies to develop COVID-19 vaccines in record time by reducing the risk for companies [22]. Both manufacturers and government-affiliated participants mentioned that governments were already engaging in pandemic preparedness activities in 2022–2023—for instance, investing in platform technologies, creating national stockpiles of specific vaccines and European Union-level tenders for an ever-warm vaccine production network (P3, P10, P11, P12). Much has been accomplished to create a better data foundation and physical infrastructure for pandemic management (P5). Some publications explore the possibility of a central vaccine “control tower,” envisioning a decision-making support tool that operates by monitoring real-time data on a broad set of indicators [45,52].

### 4.4. The Potential of mRNA Vaccines

As would any new technology under such high demand, the mRNA vaccines experienced ramp-up difficulties, since the manufacturing processes (and associated challenges) (P6) [15] and networks for capacity ramp-up (P3, P4) [18] had yet to be established. The technology bears two promising advantages: firstly, the speed at which an mRNA vaccine can be created and produced in large quantities (P12), [14] and secondly, the relative simplicity of the production process (P12), [15]. Both are great advantages over traditional vaccine types from a supply chain perspective (P4, P8). Further examinations of the full immunological effects (P2), improving vaccine stability to address cold chains (P5) [7,15], shelf-life limitations (P5, P7) [15], and finally, regulatory criteria for ensuring mRNA quality must be established [7], and efficient processes for quality assurance therefore must be developed [20].

In future health emergencies, a well-developed mRNA vaccine platform could play a crucial role in the rapid response to disease outbreaks [53]. The CEPI, for instance, has detailed a plan to roll out an RNA-based vaccine within 100 days from the identification of the pathogen by shortening the Phase 1 and 2 clinical trials based on platform data [53]. In the medium term, experts regard mRNA vaccines as a complementary technology to existing vaccine types, which have benefits and drawbacks depending on the specific pathogen and the epidemiological situation (P1, P2, P12).

## 5. Conclusions

The global scale of the COVID-19 pandemic was unprecedented, and so were the international efforts to develop a vaccine. With the increasing risk of pandemics, it is essential to learn from the challenges that have arisen. This study contributes to the existing knowledge on resilience in the VSC by capturing industry knowledge through semi-structured interviews, presented in the form of a stakeholder system overview and deep dives examining the role, constraints, and resilience from the perspective of each stakeholder.

Our qualitative study contributes to the body of knowledge serving to create more resilient VSCs. Since we approached the VSC on a system level, our findings are applicable regardless of geography; nevertheless, the local legal frameworks and infrastructural and cultural context cannot be addressed at this level. Furthermore, the insights gathered from the four stakeholders from industry (P1, P6, P7, P8) may not be representative for the entire industry. Despite this, the experiences of the different stakeholders were mostly consistent with one another, which further validates the conclusions drawn from this study. We find that the VSC is profoundly characterized by uncertainty. This uncertainty can be caused by factors external to the VSC, such as not knowing when a new emergency may arise and how the epidemiological situation will unfold. Within the VSC, there are uncertainties about the demand for vaccines, the success of vaccine candidates, competition, and the security of supply, among others. The lack of transparency between industry stakeholders perpetuates these uncertainties. In turn, the lack of overview within industry escalates to further uncertainties, affecting governmental decision-making for pandemic management. Strategic pandemic management decisions had to be made quickly despite large uncertainties, and the impact of the chosen policies on the rapid scale-up of COVID-19 vaccine production capacity remains to be assessed in detail [22]. A solution to prevent business or national interests from interfering with the best public health outcome is the installation of an overarching authority on vaccines as an efficient mediator (P6), [45]. Future management research should explore the feasibility of such an authority.

In the section on resilience, we recommend five measures to VSC businesses: (1) setting up real-time data platforms, (2) increasing supplier diversity, (3) shortening the supply chain, (4) stockpiling strategic items, and (5) building trusted networks. While uncertainty and lack of transparency are the central weaknesses limiting the functioning of the VSC, they also present a large potential for improvement. To create a more resilient system, promoting infrastructures for increased transparency and collaboration between individual stakeholders hold the greatest potential for installing resiliency in the VSC in the face of future pandemics. Decision-makers could draw on frameworks such as proactive management practices for building these trusted networks, thereby working towards transparency and collaboration [54]. Progress in this direction can improve the strategic decisions taken by individual actors and better align the actions taken by individual stakeholders for enhanced system performance.

Implementing the lessons learned from the COVID-19 pandemic will require efforts from all stakeholders and will depend on their willingness to collaborate to instill more resilience in the VSC. As a consequence of this pandemic, the awareness of the threat posed by pandemics and the benefits of vaccines has increased. These considerations will weigh against the motivations for nationalistic policies and overly protective business strategies.

## Figures and Tables

**Table 1:** Overview of interview partners (anonymized). In nine separate interviews we spoke to 12 participants along the VSC. Stakeholders are contextualized as “upstream” and “downstream” relative to vaccine manufacturing.

No.	Description of Participants	Stakeholder Group	Limitations
(P1)	Medical affairs, manager	Vaccine developer and manufacturer (large integrated company)	Group interview
(P2)	Scientific director at a research institute, advisor to a government body	Research institute/Advisory body—upstream	Group interview
(P3) (P4)	COVID-19 vaccine manufacturing COVID-19 vaccine manufacturing, manager	Advisory body/Purchaser (Government entity)—upstream	
(P5)	Immunization supply chain, manager	Purchaser (NGO)–upstream and downstream	
(P6)	Customer support, manager	Vaccine capital equipment supplier–upstream	
(P7)	Procurement, manager	Vaccine developer and manufacturer (small integrated company)	
(P8)	Procurement, manager	Vaccine manufacturer (CMO)	
(P9) (P10) (P11)	Vaccine supply interruptions, coordinator Deputy director Vaccine supply interruptions and distribution monitoring, manager	Advisory body (Government entity for pandemic mgmt.)–upstream and downstream	No recording
(P12)	Clinical development, manager	Advisory body (NGO, vaccine development funding)–upstream	

**Table 2:** Stakeholder group summary: Purchasers.

Stakeholder Group	Actors	Role and Drivers	Constraints	Resilience and Solutions
Purchasers	National governments, NGOs, private sector	Role: Purchase vaccines, generate demand, enable distribution Drivers: Public health, disease control	(Lack of) transparency for planning, Geopolitical matters (competition for resources)	Risk sharing, matching, and facilitating, improving physical and digital infrastructure, stockpiling

**Table 3:** Stakeholder group summary: Vaccine manufacturers.

Stakeholder Group	Actors	Role and Drivers	Constraints	Resilience and Solutions
Vaccine manufacturers	Large pharmaceutical companies, few small players, many CMOs	Role: Vaccine manufacturing at high quality and safety standards Drivers: Efficiency and profitability	Supply and regulations (long lead times, qualification process for additional suppliers), Quality and process (quality assurance capacities, fragmentation of the manufacturing process), Technology transfer and information (building processes, corporate knowledge, skilled labor, intellectual property, secrecy)	Standardization to facilitate qualification of suppliers and input materials, dual sourcing, regionalization, in-house production, increase transparency, with the help of digital tools

**Table 4:** Stakeholder group summary: Manufacturing input suppliers.

Stakeholder Group	Actors	Role and Drivers	Constraints	Resilience and Solutions
Manufacturing input suppliers	Few large suppliers, many smaller companies	Role: Supply of raw materials, vaccine capital equipment, fill-and-finish capital equipment Drivers: Efficiency and profitability	Lead times (constrained by capacity utilization and component availability), Demand fluctuations (volatile in pandemic situation), Geopolitical matters (inability to export), Systemic effects (production of other medicines, suppliers higher up in VSC)	Standardization, regionalization, regulatory flexibility, ensure that financial risks taken by suppliers do not impede their willingness to respond to emergencies

**Table 5:** Stakeholder group summary: Vaccine developers.

Stakeholder Group	Actors	Role and Drivers	Constraints	Resilience and Solutions
Vaccine developers	Large pharmaceutical companies, biotech companies, and academia	Role: Development of safe and effective vaccines Drivers: Profitability, public health	High investment with uncertainty of gaining approval	Fund development of vaccines against most dangerous pathogens to correct market failures

**Table 6:** Stakeholder group summary: (Inter-)national regulatory agencies.

Stakeholder Group	Actors	Role and Drivers	Constraints	Resilience and Solutions
(Inter-)National regulatory agencies	EMA, FDA, their international equivalents	Role: Issue market authorization of vaccine candidates, ensure quality and safetyDrivers: Public health	Highest quality and safety standards, Time	Harmonization of regulatory requirements

## Data Availability

Data are available upon request from the authors.

## Appendix A

**Table A1:** The ten most frequently mentioned codes; For each code, the number of occurrences and its affiliation with code groups is displayed, sorted by frequency (decreasing).

Code	Occurrences	Groups
○ stakeholders	69	Political, Social
○ supply chain	64	Supply chain resilience
○ constraints	56	Economic, Political
○ pandemic	51	Environmental
○ business model	42	Economic, Supply chain resilience
○ solutions	42	Economic, Political, Solutions
○ production capacity	41	Economic
○ regulatory	41	Legal
○ geographical differences	39	Economic, Political
○ approval	38	Legal

**Table A2:** Condensed overview of the results presented on the five stakeholder groups central to vaccine manufacturing in emergencies.

Stakeholder Group	Actors	Role and Drivers	Constraints	Resilience and Solutions
Purchasers	National governments, NGOs, private sector	Role: Purchase vaccines, generate demand, enable distributionDrivers: Public health, disease control	(Lack of) transparency for planning, Geopolitical matters (competition for resources)	Risk sharing, matching, and facilitating, improving physical and digital infrastructure, stockpiling
Vaccine manufacturers	Large pharmaceutical companies, few small players, many CMOs	Role: Vaccine manufacturing at high quality and safety standards Drivers: Efficiency and profitability	Supply and regulations (long lead times, qualification process for additional suppliers), Quality and process (quality assurance capacities, fragmentation of the manufacturing process), Technology transfer and information (building processes, corporate knowledge, skilled labor, intellectual property, secrecy)	Standardization to facilitate qualification of suppliers and input materials, dual sourcing, regionalization, in-house production, increase transparency, with the help of digital tools
Manufacturing input suppliers	Few large suppliers, many smaller companies	Role: Supply of raw materials, vaccine capital equipment, fill-and-finish capital equipment Drivers: Efficiency and profitability	Lead times (constrained by capacity utilization and component availability), Demand fluctuations (volatile in pandemic situation), Geopolitical matters (inability to export), Systemic effects (production of other medicines, suppliers higher up in VSC)	Standardization, regionalization, regulatory flexibility, ensure that financial risks taken by suppliers do not impede their willingness to respond to emergencies
Vaccine developers	Large pharmaceutical companies, biotech companies, and academia	Role: Development of safe and effective vaccines Drivers: Profitability, public health	High investment with uncertainty of gaining approval	Fund development of vaccines against most dangerous pathogens to correct market failures
Regulatory agencies	EMA, FDA, their international equivalents	Role: Issue market authorization of vaccine candidates, ensure quality and safety Drivers: Public health	Highest quality and safety standards, Time	Harmonization of regulatory requirements
